# An Integrative Framework for Bayesian Variable Selection with Informative Priors for Identifying Genes and Pathways

**DOI:** 10.1371/journal.pone.0067672

**Published:** 2013-07-03

**Authors:** Bin Peng, Dianwen Zhu, Bradley P. Ander, Xiaoshuai Zhang, Fuzhong Xue, Frank R. Sharp, Xiaowei Yang

**Affiliations:** 1 Department of Health Statistics, Chongqing Medical University, Chongqing, China; 2 Division of Biostatistics, Bayessoft, Inc., Davis, California, United States of America; 3 Hunter College–School of Public Health, City University of New York, New York, United States of America; 4 Medical Investigation of Neurodevelopmental Disorders (MIND) Institute, University of California Davis, Sacramento, California, United States of America; 5 School of Public Health, Shandong University, Jinan, Shandong, China; University of California Riverside, United States of America

## Abstract

The discovery of genetic or genomic markers plays a central role in the development of personalized medicine. A notable challenge exists when dealing with the high dimensionality of the data sets, as thousands of genes or millions of genetic variants are collected on a relatively small number of subjects. Traditional gene-wise selection methods using univariate analyses face difficulty to incorporate correlational, structural, or functional structures amongst the molecular measures. For microarray gene expression data, we first summarize solutions in dealing with ‘large p, small n’ problems, and then propose an integrative Bayesian variable selection (iBVS) framework for simultaneously identifying causal or marker genes and regulatory pathways. A novel partial least squares (PLS) g-prior for iBVS is developed to allow the incorporation of prior knowledge on gene-gene interactions or functional relationships. From the point view of systems biology, iBVS enables user to directly target the joint effects of multiple genes and pathways in a hierarchical modeling diagram to predict disease status or phenotype. The estimated posterior selection probabilities offer probabilitic and biological interpretations. Both simulated data and a set of microarray data in predicting stroke status are used in validating the performance of iBVS in a Probit model with binary outcomes. iBVS offers a general framework for effective discovery of various molecular biomarkers by combining data-based statistics and knowledge-based priors. Guidelines on making posterior inferences, determining Bayesian significance levels, and improving computational efficiencies are also discussed.

## Introduction

Biomarkers play a central role in the development and conduct of translational and personalized medicine [1]. They are used in predicting the progression of disease (prognosis markers), selecting treatment regimes (predictive markers), screening diseases (diagnostic markers), and assisting with other forms of health related tasks. Genomic biomarkers have already been applied for making critical decisions, e.g., the Oncotype Dx test for quantifying risk of disease recurrence in women with early-stage breast cancer and for assessing the likely benefit from certain types of chemotherapy [2]. The most notable challenge in molecular biomarker discovery is caused by high-dimensionality of the data sets. There are thousands of genes in microarray data analysis [3] and millions of single nucleotide polymorphisms (SNPs) in genome-wide association studies (GWAS) [4] from which biomarkers are identified.

Traditionally, discovery of differential genes was achieved by univariate analyses where each gene is considered individually, e.g., the weighted voting scheme of Golub et al. [5], the partial least squares of Nguyen et al. [6], and the Wilcoxon test statistic of Dettleing et al. [7]. Such gene-wise comparison methods have to deal with the multiple comparison problem. Although schemes have been proposed in adjusting the study-wise type-1 error or restraining false positive rates, there lacks an effective way to explicitly incorporate correlational or functional relationships between the genes. Without studying the interactions of genes and their joint impacts on phenotype, the traditional gene-wise methods barely offer any biological interpretation. An earlier trial to link gene-wise tests together was seen in LIMMA [8] using the idea of empirical Bayes [9]. It makes the analysis stable by borrowing information across genes via Bayesian hierarchical modeling and shrinkage estimator [10]. Similar to gene-wise analyses, LIMMA still treats gene expressions as outcome variables and compares them across experimental conditions.

A more straightforward approach is to treat disease status or phenotype as the outcome variable while setting genes as predictors. This arrangement is not only meaningful, but allows for studying multiple genes’ joint impact on the outcome variable. Thereby, the task of biomarker identification naturally becomes a problem of variable selection in fitting regression models. Standard frequentist or likelihood-based variable selection schemes via criterion assessment such as BIC and AIC or stepwise subset selection algorithms become infeasible when the number of variables 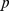 becomes large; see an extensive discussion in Miller [11]. As an alternative solution, Bayesian variable selection (BVS) not only provides intuitive probabilistic interpretation, but also explores the model space efficiently in a stochastic way to ensure that the models with high probabilities would show up earlier and more frequently during a simulation process. This is the reason that the first satisfactory scheme of BVS was called ‘stochastic search variable selection (SSVS)’ [12]. The theory of SSVS was further developed [13] and many other stochastic searching schemes have been proposed, e.g., the simplified method of Kuo and Mallick [14], the Gibbs variable selection [15], Geweke’s BVS with block-updates [16], and the reverse jump MCMC algorithm [17]. BVS algorithms were also extended to much wider settings, e.g., generalized linear models (GLMs) [18], [19]; multivariate regression models [20]; and even mixed-effects models [21], [22]; see O’Hara and Sillanpää [23] for a detailed review.

The first applications of BVS in the setting of 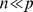 arose from analyzing genetic data in the early 2000s. Examples include Bayesian model selection in gene mapping studies [24], [25] and SSVS for identifying multiple quantitative trait loci [26]–[28]. Most of the methods use hierarchical Bayesian modeling to enable borrowing information from neighbors [29]. It is especially noteworthy that BVS has been successfully applied to GWAS data that contains millions of genetic variants or SNPs [30], [31]. As stated by Guan and Stephens [4], “even using relatively simple MCMC algorithms, BVS can indeed produce useful inferences in problems of this size (with thousands of SNPs)." For genomic data, including genome sequencing and gene expression microarray data, biomarker identification with full BVS strategies is becoming popular. BVS resorts to hierarchical modeling to control the model size while as much as possible allowing data structures to be complex [32], [33]. A fair number of BVS applications have been demonstrated in the previous decade [34]–[36].

A recent focus in BVS development is on how to model biological processes that involve gene or protein groups functioning in concert. A comprehensive understanding of such processes would help to unravel disease mechanisms and to design more effective therapeutical products [37], [38]. Recent studies demonstrate that evaluating changes in expression across pre-defined gene sets often increases statistical power and produces more robust results [8], [39]–[42]. Therefore, an appropriate approach eliciting biologically meaningful and informative priors for BVS is a worthy pursuit [38], [43], [44].

Following the above review of BVS development history, a generalized strategy called integrative BVS (iBVS) for biomarker discovery is presented in the Methods section. We propose an iBVS strategy with a novel prior called PLS g-prior for handling covariance matrices with 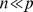 and incorporating gene pathways into the selection procedure. In the Simulation section, the above iBVS for gene expression data with binary disease status is validated using simulated data and compared with other standard BVS routine. In the Application section, the strategy of iBVS is illustrated using a practical Affymetrix microarray data set for patients with stroke. Remarks and discussions are given in the Discussion section.

## Methods

### Notations

Suppose that 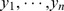 are 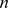 independent observations of the outcome variable 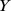, which could be binary, count or continuous. Each outcome is associated with a set of predictor variables 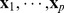 whose values form the matrix 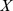:
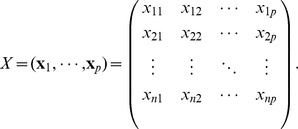



In microarray data, 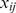 denotes the normalized level of expression for the 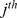 gene on the 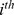 subject. The outcome variable 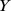 is said to have a distribution in the *exponential family* if its probability density function can be written in the general form, 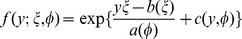. Most of the distributions that we know such as Gamma, Beta, Poisson, and Gaussian distributions are all members of the exponential family. When 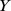 follows an *exponential family* distribution, the GLM [45] is introduced in studying the relationship between 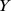 and 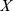 via
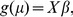
(1)where 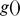 is a *link function* after which the expected value of 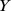, 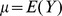, is predicted by the linear combination of 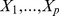.

### Bayesian Variable Selection in GLMs

A fundamental task of regression analysis is to select which subset of the predictors are used to predict or explain the variance 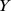. When other features of the GLMs such as the choice of link function are determined, the problem of variable selection is equivalent to the task of model selection. This paper focuses on the explicit way of Bayesian variable/model selection in which an indicator vector 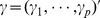 is introduced where
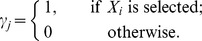



Then the equation of GLM can be rewritten as
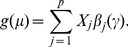
(2)


By specifying prior distribution of 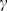 and 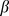 (and possibly other parameters such as residual variance in a linear regression model), one applies Bayes rule to derive the posterior distribution 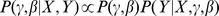 from which to obtain the posterior probabilities 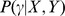 for model selection (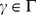). Within the scope of this article, we define BVS as a procedure of variable selection based on the posterior marginal selection probabilities,
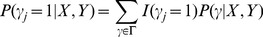
(3)which has the form of Bayesian model averaging (BMA) [46]. This BVS selection probability 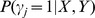 calibrates the overall strength of 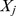 in predicting 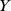 across various models.

Depending on the specification the prior distribution, 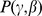, many schemes of BVS have been proposed, e.g., independent prior distributions (i.e., 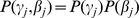) [14] and Gibbs variable selection where 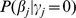 is set as a ‘pseudo-prior’ [15]. The most influential scheme is the SSVS [12], which assumes that 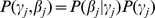 with
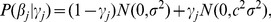
(4)


a mixture of a concentrated Gaussian distribution (when 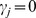) and a diffused one (when 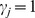 and 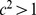). Alternatively one may specify 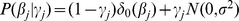, which has a natural interpretation and can be further extended to the multivariate setting, i.e., the g-prior [47],
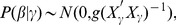
(5)where 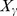 is the sub-matrix of 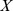 consisting of columns with 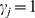, and constant 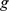 can be fixed at 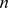 or estimated via empirical Bayes. The g-prior is a conjugate prior; one may analytically integrate out 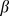 from 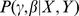 to obtain 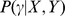 or 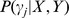
's, which are of primary interest in BVS.

Nonetheless, g-prior has an undesirable feature: as 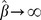, 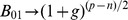, where 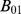 is the Bayes factor in favor of the null model (i.e., the one with 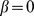). It is because of this Bayes factor paradox that Jeffreys [48] rejected normal priors, and later Zellner and Siow (ZS) [49] proposed the Cauchy prior,
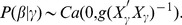
(6)


From the viewpoint of objective Bayes [50], ZS-prior satisfies six of the seven desirable features (e.g., consistency, predictive matching, and invariance) for the choice of model prior, but it does not lead to closed-form answers. It was then further extended to the so-called ‘robust prior,’ which is formulated as the scale mixture of normal distributions [51]. Please see Bayarri et al. [52] and the reference therein for recent development of objective BVS priors, e.g., intrinsic priors [53]–[55], expected posterior priors [56], and integral priors [57].

The prior 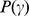 can be naturally set as 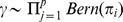. When there is no preference, we can simply let 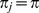, i.e., 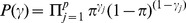. The value of 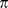 can be set to control the number of selected variables a priori. For a data set with 100 variable, setting 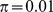 implies that only one variable be selected before observing the data. We do not recommend using 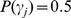 because it indicate equal probabilities (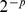) for all models and does not induce any multiplicity adjustment [58]. Alternatively, one may introduce a hyper prior distribution for 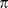, 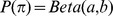, which could provide automatic multiplicity adjustment [59]–[61].

### BVS When 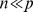


Note that most of the above discussion assumes 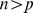. When BVS is applied to biomarker identification for genomic data where it is often seen that 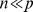, we face many challenges. First, since the size of model space (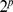) increases exponentially with 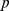, it becomes an intimidating task for a thorough search among all genes. Even with stochastic searching strategy, the MCMC sampling algorithm has a large computational burden at the level 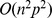. Second, among the 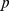 genes, many of them are ‘noisy’ variables in the sense that they either have low quality such as missing or censored values or do not participate the biological processes under study. Blindly including them into the analysis would make the modeling procedure time consuming or end up with invalid conclusions. Third, the rank of matrix, 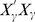, would be much smaller than 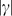, the number of selected genes, making the matrix inversion impossible. Fourth, there are many genes whose expressions are highly correlated, easily leading to singularities in setting priors as well as deriving the posterior distributions. These between-gene correlation or causal structures, on the other hand, cannot be simply ignored.

**iBVS–A Generalized Framework of BVS.** To solve the above problems, we could have two options: (1) to restrain the size of model space to a level that BVS can be accomplished within acceptable amount of time; and (2) to apply the principle of parsimony to reduce the number of model parameters via regularization and shrinkage estimators. In this article, we provide a generalized 2-step procedure called iBVS.

**Step One** is a ‘robust’ screening process aiming to directly reduce the dimension 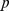 by removing genes with little useful signal or those having no known biological relationship to the target disease or phenotype. By ‘robust’ screening, we mean to use the combination of various criteria jointly to ensure that enough genes are included for Step Two. For example, we can first conduct gene-wise t-tests to remove genes with p-values larger than a pre-specified cut-off level (e.g., 0.01) that is much higher than the level after a multiplicity adjustment. Among the excluded genes, we may conduct gene-wise Wilcoxon test to further verify that no gene has a p-value smaller than 0.05; otherwise the genes will be moved back into the gene set. We may also move back those genes that have been discovered to be functionally or structurally related to the target disease or phenotype in the study. By curating public data bases, we can generate a list of proteins that are functionally related to the 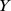, and then find all the genes that code them. All these genes would be moved back to the gene set for consideration. We should also further move back additional genes that are connected to any genes in the current set according to a specific way of defining gene-gene networks, e.g., metabolic pathways [62] and protein-protein interaction networks [63]. The final retained set of genes is termed as the ‘signature set’ and used for Step Two. The screening process may also allow investigators' subjective preferences and other methods such as the *topological analysis of co-expression network* [64] and *bagged gene shaving* [65]. Here, we emphasize that the screening is not purely just for dimension reduction based on testing statistics. It is rather a key component of iBVS for biomarker identification, which aims to create a broad enough but biologically meaningful signature set for further conducting BVS in the next step.

**Step-Two** of iBVS focuses on variable selection within the framework of Bayesian hierarchical modeling (BHM) that aims to investigate the joint distribution of genes in the signature set in predicting phenotype or disease status. BHM offers a flexible way in modeling complex structured data while restraining the number of parameters. To reduce the computation burden of BVS for large 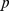 (number of signature genes), various strategies are conceived. As seen in Godsill [66], and Yi [67], we can adopt the ‘composite model selection’ principle and restrict that in each MCMC iteration, only models with 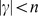 are allowed to be selected. This can be done by creating a special proposal distribution in the Metropolis-Hastings (M-H) algorithm. Using the idea of ‘Leaps and Bounds’ [68], Annest and Bumgarner et al. [69] proposed the iterative model selection algorithm that first orders all variables with a univariate selection method and then moves a 30-variable window down the ordered variables. To handle the problem of 
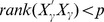
, a direct solution is by Yang and Song's 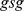-prior [70], which is the generalized inverse of 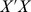 in Zellner's g-prior. Ridge regression is also originated to handle the problem of inverting a nearly singular matrix and Cholesky decomposition is usually adopted to speed up the matrix inversion. Some high-performance Gibbs samplers and M-H sampling algorithms have been developed [71]. A straightforward solution is to run multiple chains simultaneously (see Gelman and Rubin [72]) on multiple virtual machines in computer clusters or using Cloud Computing platforms. Some approximation methods are also introduced trying to improve the computing speed, e.g., the Matching Pursuit method [73], [74].

In this paper, we proposed an iBVS with novel prior called PLS g-prior in dealing with large 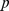 problems and with informative prior on variable selection that reflect the gene-gene networks using Markov random field (MRF).

### Incorporate Informative Priors

Meaningful prior information may come from different sources, including published literature, online knowledge bases, and empirical experience of the investigators. However, it is still not completely clear how to best use them or relate them effectively in variable selection [75]. The most convenient way for informative prior elicitation is to incorporate the relative frequencies of identified biomarkers from published literature or from investigators' subjective preference. For example, Kitchen et al. [76] used results from the scientific literature when constructing several informative exchangeable subset selection priors.

A more comprehensive approach is by adopting the view of systems biology, which studies biological processes as whole systems instead of isolate parts. For many diseases, expression-based classification alone do not achieve high accuracy because changes in expression of the few genes causing disease can be subtle compared to those of the downstream effectors, which vary considerably from patient to patient. A more effective means of marker identification is to combine expression measurements over pathways and identify which pathways act as markers in predicting or explaining phenotypes. Here pathway refers to a group of functionally or structurally related genes that jointly form a network. Several pathway- or network-based marker identification approaches have been proposed recently, e.g., Chuang et al. [77] integrates expression profiles with pathways extracted from protein interaction networks and Lee et al. [78] does so by adopting pathways curated from literature. Large protein-protein interaction networks have recently become available for human, enabling new opportunities for elucidating pathways involved in major diseases and pathologies. This network-based marker discovery approach has shown success in diagnosis of metastatic breast cancer [77] as well as classification of cell fate decisions during development [79].

In this article, we combine the idea of gene- and network-based marker discovery and provides an iBVS framework for identifying contributive genes and important pathways. Informative priors on pathway definition could come from publicly available literature and databases: (1) DNA-sequence data (e.g., GeneBank and EBI); (2) RNA sequence data (e.g., NCBI and Rfam); (3) GWAS data (e.g., dbSNP and HapMap); (4) protein sequence data (e.g., UniProt, PIR and RefSeq); (5) protein class and classification (e.g., Pfam, IntDom, and GO); (6) gene structural (e.g., ChEBI, KEGG ligand Database, and PDB); (7) genomics (e.g., Entrez Gene, KEGG, and MetaCyc); (8) Signaling pathway (e.g., ChemProt and Reactome); (9) metabolomics (e.g., BioCycy, HMDB, and MMCD); (10) protein-protein interaction (e.g., IntAct, DIP, MiMI). These databases could help us define pathways or networks upon which to map our gene expression data under analysis. Using the available biological information on inter-connectivities and interactions between genes, we aim to discover pathways that are associated with a specific biological process. Srivastava et al. [80] have employed the GO information into their priors, and Stingo et al. [38] have used KEGG network information.

There are also many other types of public data that may not be used directly to construct pathways, but could be used directly for deriving prior distributions for the current model. For example, by searching literature (PubMed and Google Scholar) or reanalyzing older gene expression data from GEO, ArrayExpresss, and Oncomine, we could have some insights in determining the size and form of the model before analyzing the data set at hand. Many available clinical (e.g., OMIM, GeneCards, and CancerGenes) or drug databases (e.g., DrugBank and SuperTarget) could also provide structural or semi-structural information for us to restrain model space and parameters.

### Posterior Inference

As was mentioned in section 1, when the posterior space is huge, we usually use the MCMC simulation to fit the posterior distribution [81], instead of trying to obtain the exact values via complicated calculations. The Gibbs sampler, and M-H sampler are some of the well known Markov Chain Monte Carlo (MCMC) algorithms. If possible, one should first analytically integrate out the nuisance parameters (e.g., 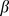, which is not of our main interest). This can significantly speed up the MCMC simulation procedure. As seen later, there are other ways to enhance the speed and efficiency of MCMC, including various means to define the proposal function in an M-H algorithm.

Once we obtain the samples from a MCMC procedure, we can summarize them to estimate the posterior probabilities of selecting genes and selecting pathways. In practice, certain guidelines should be followed for making posterior inferences. The setting of a cut-off for the important genes and pathways should adopt the cross-validation strategy. As many research papers have shown, in comparison to choosing one single best model, Bayesian model averaging (BMA) would provide a better performance in prediction problems [82]. It is also possible that different models be used for variable selection and for making prediction or classifying samples using the selected variables; model selecting and making predictions are often viewed as two different goals.

### iBVS for Biomarker Identification with Binary Outcome

In this section, we illustrate our iBVS method for biomarker identification for gene expression data with binary outcomes. Here we employ Bayesian hierarchical modeling approach to do gene selection and pathway selection simultaneously and the PLS g-prior is introduced.

Suppose our gene expression data have up to 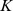 pathways, we denote the pathway membership by the matrix 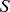 (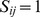 if the 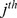 gene belongs to 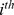 pathway; 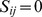 otherwise), and denote gene-gene network by the matrix 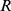 (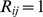 if there is a direct edge between the 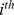 and 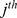 genes; 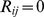 otherwise). In addition to using the indicator 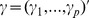 for gene selection, we introduce another indicator 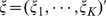 for pathway selection, where 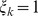 (or 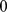) if the 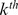 pathway is selected (or excluded). When the outcome variable 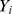 is binary, the Probit model of Albert and Chib [83] is applied,
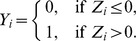
where the latent variable 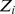 is assumed to have the standard normal distribution, i.e.,
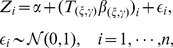
(7)


where 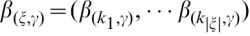, 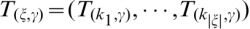, with 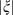 denotes the number of selected pathways in predicting 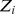, and 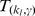 denotes the vector of first PLS component of 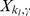. Note that 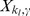 is the sub-matrix of 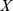, consisting of only the columns that correspond to selected genes in the selected 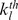 pathway. Here, we use 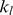 (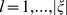) to index the number of the 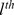 selected pathway (i.e., 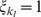); e.g., when 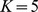 and 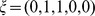 is the pathway selection result, we have 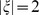, 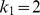, and 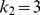.

#### Prior specification for regression parameters

Note that since observation data 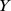 and expression data 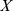 are usually standardized, we'll assume 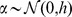, where 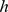 is usually chosen as a large number to indicate that we have little prior information on the value of 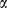. As for the prior on 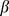, some commonly used priors include a mixture distribution of a two normals, one normal and one point mass, or one point mass and one uniform; Zellner's 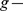
priors, Zellner-Siow's Cauchy priors, or equivalently a mixture of infinitely many normals. Yang and Song [70] generalized 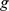-prior to the so called 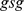-prior.

For our hierarchical model, we propose a generalized g-prior called PLS g-prior,
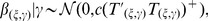
(8)where 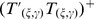 represents the Moore-Penrose generalized inverse of 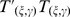, similar to Yang and Song [70]. The name comes from the fact that 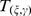 is the first PLS component of 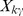. Note that this generalized inverse is well-defined for any matrix.

#### Prior specification for variable selection indicators

Following the principles in setting priors on variable selection indicators, we assume that the pathway selection indicators 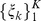 are independently Bernoulli distributed,
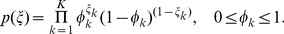
(9)where 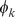 indicates the proportion of pathways expected a priori in the model. One may assume that 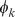 follows a mixture distribution of a Dirac Delta distribution and a Beta distribution: 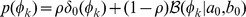. If we integrate out the hyper-parameters, 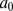 and 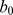, to get the marginal distribution of 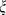, we will end up with a product of Bernoulli distributions with parameter 
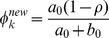
. Later on, we will omit the “new” in superscript and still denote it as 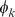.

To take into account the pathway membership information for each gene as well as the biological relationships between genes within pathways as indicated by the matrix 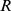, we follow Li and Zhang [43] and Stingo et al. [38] and use a MRF to describe the prior on each component of the gene selection indicator 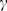,
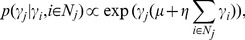
(10)where 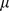 and 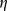 are tuning parameters that will be specified later, and 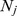 is the set of neighbors of gene 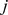 within the selected pathway. This is equivalent to the multivariate form 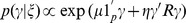; here the 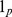 is the vector consisting of 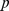 1's. There are other ways to take advantage of the MRF information too, e.g., Wei and Li [84] took a form similar to 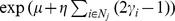 to take into account the possible down-regulating effect from neighbors.

In the above hierarchical model, we also need to include constraints on 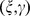 so that (a) no empty pathways will be included; (b) no gene will be selected unless the pathway containing it is already selected; (c) adding or removing genes will not cause two selected pathways having identical sets of selected genes. Any violation to these three constraints will lead to an invalid configuration. Thus, we end up with the joint distribution of 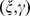 in the following form, except for invalid configurations where 0 probability will be assigned:
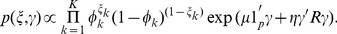
(11)


#### Derivation of posterior distributions

The joint posterior distribution of 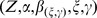 given 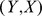 is.
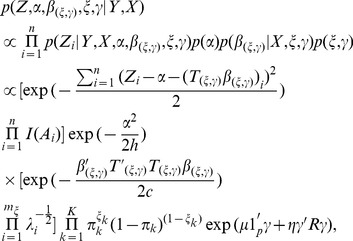
(12)


where 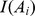 is the indicator function and 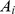 is either 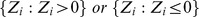 corresponding to 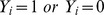, and 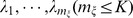 are the nonzero eigenvalues of 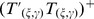.

We integrate out 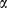 and 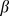 to obtain the joint posterior distribution of 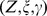 as follows (See Text S1 for detailed derivation):
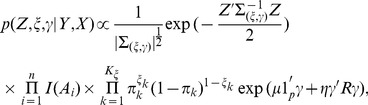
(13)where 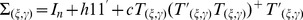.

### Computation with MCMC Algorithms

To sample the posterior distribution, we use a hybrid Gibbs and Metropolis-Hastings MCMC sampling technique, which consists of the following:

(a) Sampling 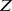 given 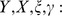 We can see from (13) that
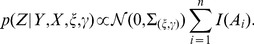
(14)


In this article, we follow the method given in Devroye (1986) to sample each element 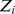 from its univariate truncated normal distribution 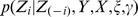, where 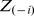 is the vector of 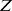 without the 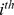 element.

(b) Sampling 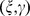 from 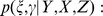


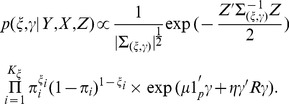
(15)


The parameters 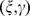 are updated using a Metropolis-Hastings algorithm in a two-stage sampling scheme. The pathway-gene relationships are used to structure the moves and account for the constraints specified earlier. Details of the MCMC moves to update 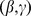 are similar to that given in Stingo et al. [38] and consist of randomly choosing one of the following random move types that will not give rise to invalid configurations as seen earlier.

## Simulation Studies

### Study Design

To verify the performance of iBVS and compare it with other methods, we mainly conducted simulation studies using KEGG pathways. First, we simulated gene expression data for 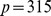 genes that involves in 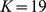 pathways as defined in the KEGG database. From the pathway structures defined, we obtained the pathway membership matrix 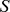 (i.e., a 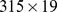 matrix; 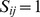 if the 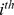 gene belongs to 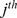 pathway, 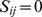 otherwise) and gene-gene connection matrix 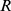 (i.e., a 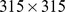 matrix; 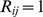 if there is a direct edge between the 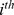 and 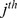 genes, 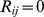 otherwise). Then we simulated the binary outcome variable, 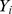, which was generated using the probit model with a latent variable 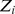. By applying iBVS algorithm with PLS g-prior to these synthesized data with know causal genes, we aimed to assess its sensitivity and specificity for gene selection. To further verify that iBVS could be applied in a practical setting with large number of genes, we also synthesized data with 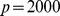. Finally, we also compared iBVS to a BVS strategy that does not employ informative priors.

**Figure 1:**
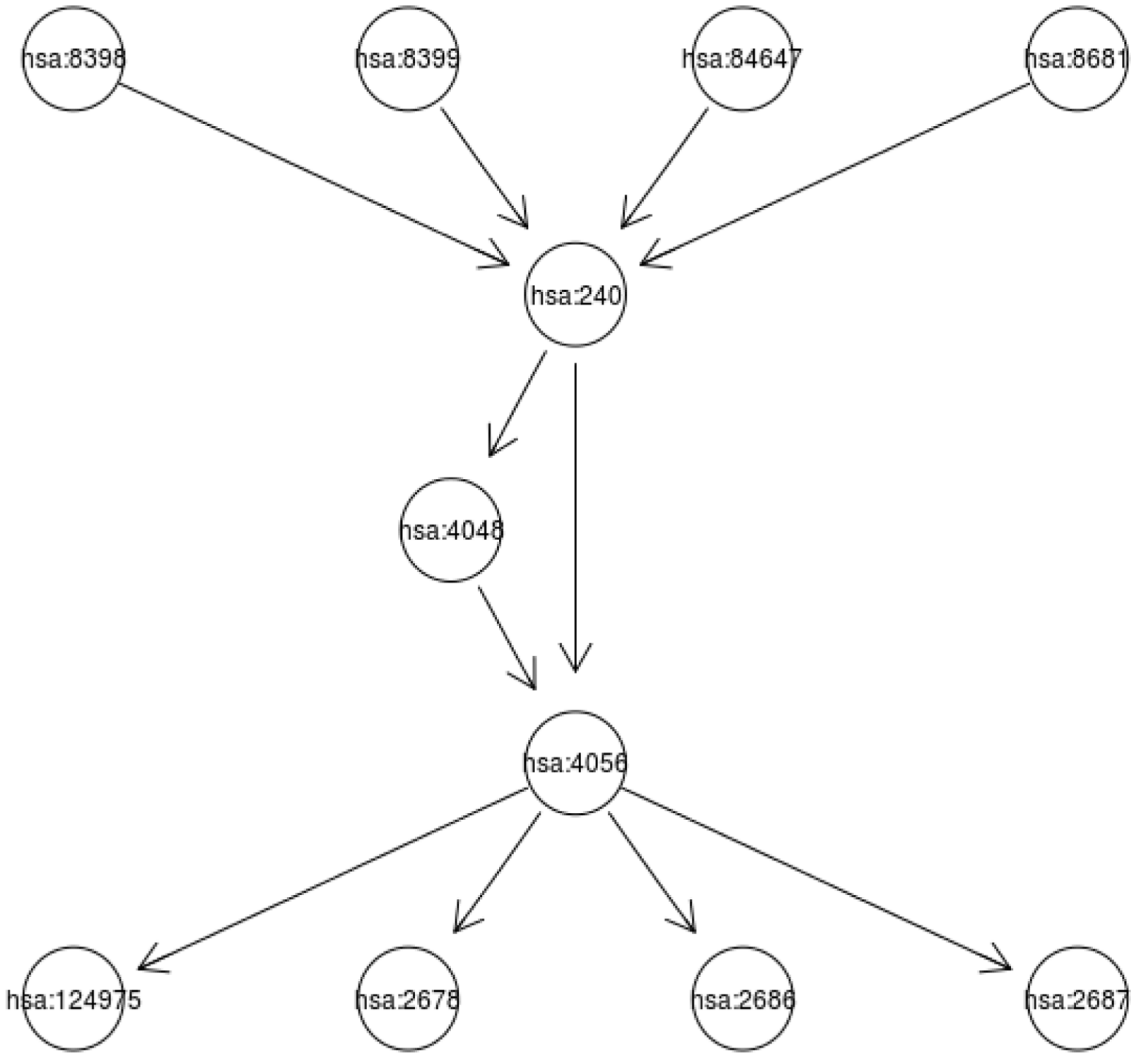
An Example of KEGG Pathway.

Each KEGG pathway can be approximately viewed as a Bayesian Network (BN), as illustrated in Figure 1. For the case of 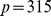, we first merged all pathways into one large 315-gene BN to take into account the genes on multiple pathways. Then we simulated expression values for all the ‘root genes’ (those without parental genes directing to them in the BN) using independent standard normal distributions. Then, the expression values of their child nodes were simulated using the idea of structural equation modeling, i.e., 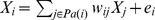, where 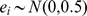 and 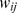
's were random weights to ensure that 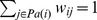. Repeating this procedure, we created 100 samples of expression values for the 315 genes. Then we standardized all the genes' expression values to ensure 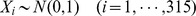.

To simulate binary outcomes, latent variables 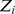
's were first simulated according to the equation 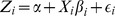, where 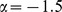 and 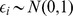 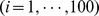. Then, the binary outcome 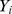 is set to be 1 if 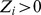, otherwise it is set to be 0. Four scenarios of simulation are conducted based on the number of causal genes and number of total genes.

### Scenario 1

For 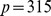, we chose only 8 causal genes: 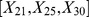 from the 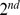 pathway; 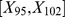 from the 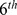 pathway; 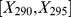 from the 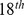 pathway; and 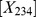 is shared by the 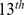 and 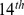 pathways. More specifically, we have.
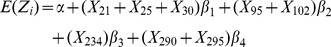
(16)


where 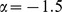 was fixed, while two levels of gene effect size (i.e., 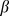 ) were considered: 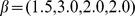 vs. 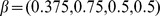. To evaluate the impact of correlation structure on gene/pathway selection, the 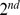 pathway was pre-specified with highly correlated member genes (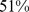 gene-gene correlation coefficients were larger 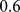 or smaller than 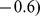, the 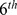 pathway with lowly correlated members (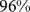 gene-gene correlation coefficients were between -0.20 and 0.20), and the 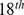 pathway with mixed high and low correlations.

### Scenario 2

Still for 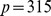, all 19 genes in the 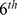 pathway and all 9 genes in the 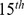 pathway were set as causal genes, i.e.,
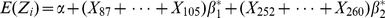
(17)where 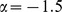 was fixed and 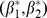 were set as 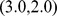 or 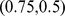. As mentioned above, the 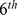 pathway mainly contains genes that are weakly correlated. The 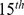 pathway has both strong and weak correlations (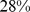 gene-gene correlation coefficients with absolute value larger than 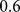; 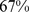 smaller than 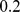).

### Scenario 3

To consider the much higher dimensional situation, we extended our simulation studies for *Scenario 1*. We kept the original sample size of 100, number of causal genes at 8, effect size 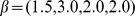, but this time added 1685 more randomly generated non-causal genes, corresponding to 81 more artificial pathways. Hence, the total number of genes in each data set is 2000, belonging to 90 pathways, and the R matrix (i.e., with elements valued at 1 or 0 to indicate pairwise gene-gene connectivity) is 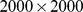.

### Scenario 4

To study the case of large grouped causal genes as seen in *Scenario 2*, we artificially set 50 causal genes from 5 pathways (i.e., genes numbered 40–49, 305–314, 950–959, 1320–1329, 1710–1719) with causal-effect set as 1.0 for the 1st 10 causal genes, 2.5 for the 2nd 10 causal genes, and similarly 1.5, 3.5, and 1.2 for other three groups. For the 1st and 2nd gene sets, the pathways they belong to were drawn from the KEGG database with many gene-gene connections; but for the 3rd, 4th, and 5th sets of causal genes, the pathways they belong to were purposedly constructed with no gene connected to other genes.

#### Parameter specification and MCMC sampler

**Figure 2:**
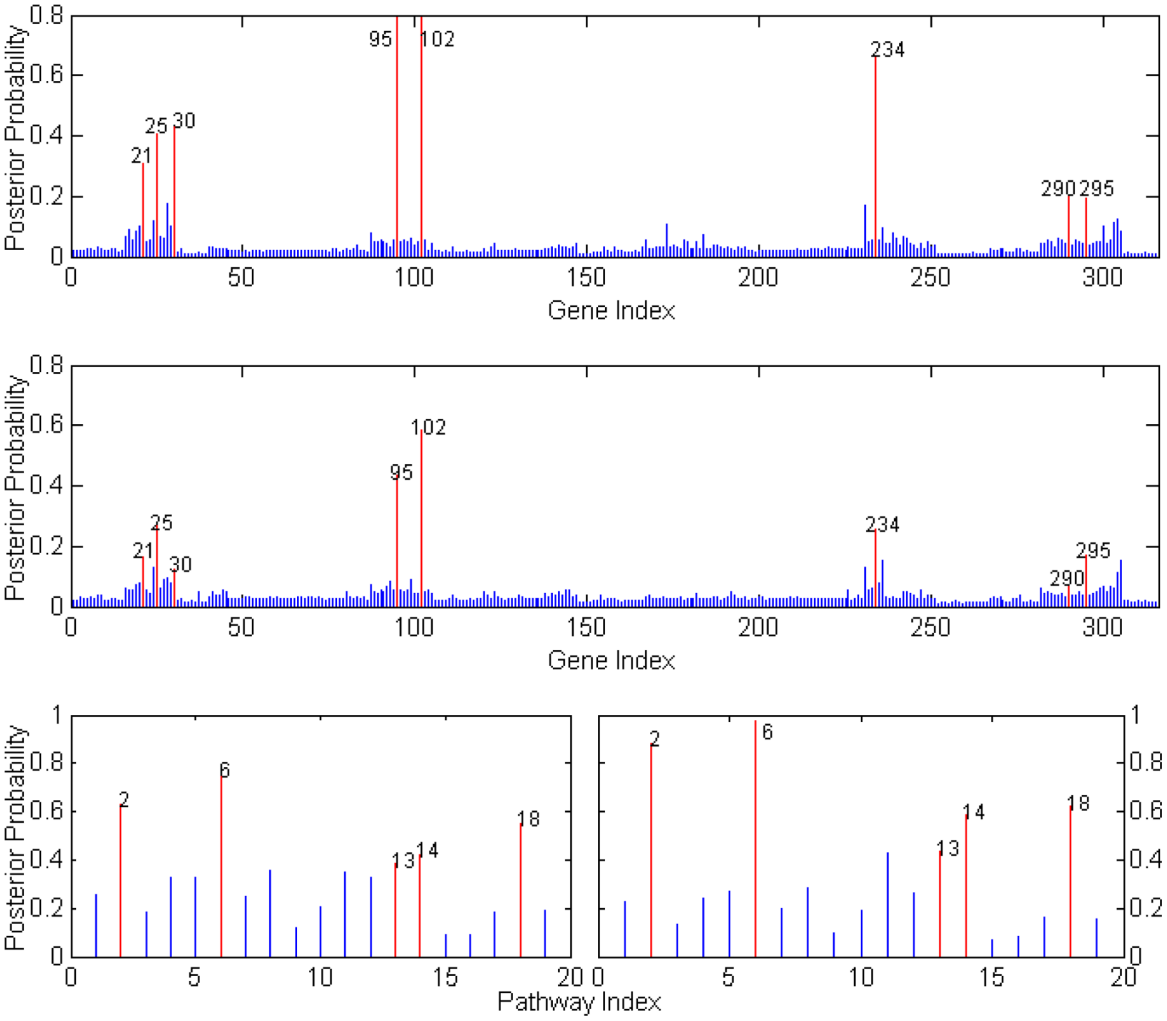
Gene and Pathway selection results in *Scenario* 1. The top figure corresponds to the posterior distribution of gene with effect size 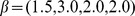, and second figure 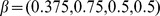. The two smaller figures on the bottom demonstrate the posterior pathway selection probabilities, with the left one corresponding to 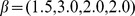, and right one 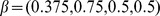. The labeled red lines indicate causal genes or causal pathways (those containing causal genes). These distributions were obtained by averaging over the 100 simulated sets of data.

**Figure 3:**
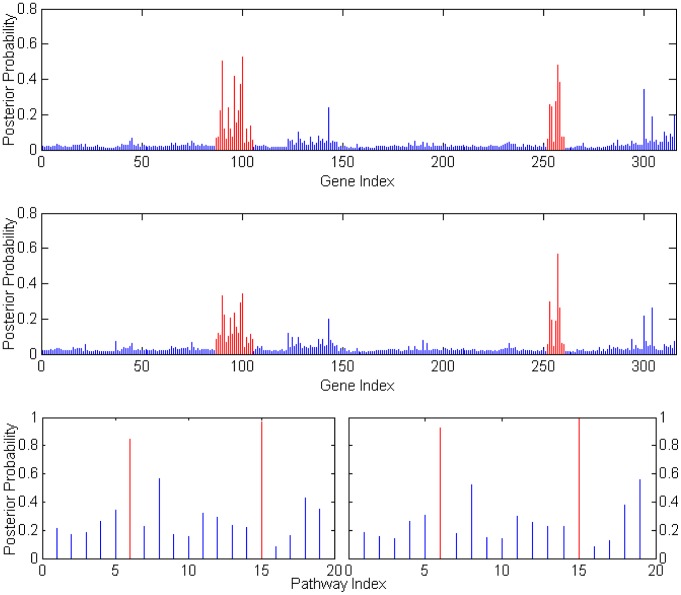
Gene and Pathway selection results in *Scenario* 2. The top figure corresponds to the posterior probabilities of gene selection with effect size 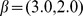, and second figure 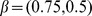. The two smaller figures on the bottom demonstrate the posterior probabilities of pathway selection, with the left one corresponds to 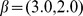, and right one 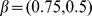. The red lines indicate causal genes or causal pathways (those containing causal genes). These distributions were obtained by averaging over the 100 simulated sets of data.

For each scenario with each choice of effect size, we repeated the above procedure to create 100 data sets, each consisting of 100 samples. Each data set was fed to the iBVS algorithm for the selection of important genes, where we set hyper parameters as 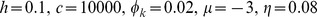. Using Gelman and Rubin diagnostics [72], the burn-in length was set at 10000 iterations after which 50000 additional iterations were run for making posterior inference on each data set. The posterior gene/pathway selection probabilities were then averaged across 100 data sets to assess the performance of BVS and the averaged selection probabilities are depicted in Figures 2 and 3. For each MCMC run on one set of data, it took about 10 minutes using a fairly fast desktop computer (Windows 7, with 4 core 2.3 GHz CPUs and 4 Gb memory). For the same task with 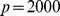, it took 54 minutes, which is still an acceptable speed.

### Simulation Results

#### Posterior selection probabilities for p = 315

In *Scenario 1*, Figure 2 depicts the posterior gene/pathway selection probabilities, averaged over the 100 simulated sets of data, for the two levels of effect sizes. The labeled red lines indicate causal genes in the left plots and the ‘causal pathways’ (those containing causal genes) in the right plots. When the gene effect size was set as 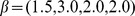, the ‘signal-to-noise ratio (SNR)’ is as high as 54.5 and it is a relatively easier task of gene selection. One observes that our iBVS with PLS g-prior did a great job; all the eight genes with the highest posterior probabilities are exactly the same preset causal genes, and the five top pathways are exactly the same causal pathways.

In comparison, when the effect size was set as 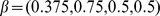, the SNR becomes 3.4, which makes it a much harder job for gene selection. Even for this challenging task, our iBVS works fairly well. Although some non-causal genes' averaged selection probabilities stand out, even higher than those of several causal genes, these non-causal genes are meaningful markers in the sense that they belong to causal pathways and are highly correlated with the causal genes. For example, 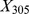 has a higher selection probability than 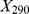, but it belongs to a causal pathway (the 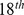 pathway) and has a correlation coefficient of 0.86 with the causal gene 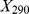. For two highly-correlated genes within one pathway, it does not make much difference which one is selected to act as the ‘marker’ in the conduct of personalized medicine.

As for influence of correlation structures, first in the 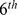 pathway, genes are weakly correlated, hence the causal genes are clearly selected out. In the 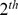 pathway, genes are highly correlated, we see that non-causal genes also have relatively high posterior selection probabilities and the cut between causal and non-causal is not that clear. This is especially seen in the case with smaller effect size. As one expects, the contribution of a pathway in predicting the outcome 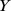 should be determined not only by the effect sizes of causal genes in it, but also by the number of causal genes in it. This is exactly the result observed from the iBVS strategy. For example, gene 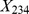 is a causal gene, belonging to both the 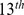 and 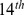 pathways. Hence we see that both pathways stand out from non-causal pathways, but at the same time their averaged posterior selection probabilities are lower than that of the 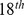 pathway because the latter has two cause genes (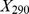 and 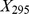), each having equal effect size with 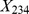.

In *Scenario 2* all genes in pathways 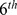 and 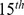 are causal genes. Plots in Figure 3 clearly show that the two groups of genes tend to have higher posterior probabilities whether the effects of causal genes are high or low. It is even clear that the top two causal pathways stand much higher above the rest in terms of posterior selection probabilities. Comparing the 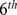 and 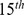 pathways, it is seen that the former has a relatively lower pathway selection probability, although it has larger number of genes and each of the gens has stronger caul effect (i.e., 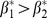). An interpretation is that the 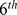 pathway contains genes that are highly correlated; hence the effective degrees of freedom is smaller than that of the 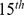 pathway. Once again, this proves that not only the number of causal genes, but the correlation structure between genes would affect the selection of pathway in predicting disease or phenotype.

It is also noted that pathways 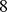 and 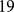 and their member genes tend to have higher selection probabilities as seen from the plots. This is because some of the genes in these two pathways are highly correlated with some of the causal genes in the 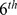 and 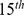 genes. The higher absolute level a non-causal gene is correlated with some causal genes, the higher the posterior selection probability would be observed for it. This is also the reason that in practical settings, marker genes instead of causal genes are often identified. We also found that the direction of the correlation coefficient would not affect the selection probability of a marker gene; that is, the correlation coefficient of 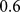 or 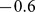 between non-causal gene A and the causal gene B would lead to the same increase of A's selection probability.

#### Posterior selection results for P = 2000

**Figure 4:**
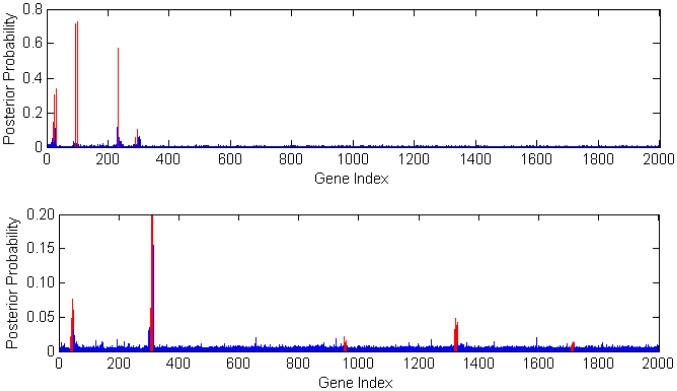
Posterior Gene Selection Probabilities when P = 2000. The top figure shows the result for *Scenario* 3, and the bottom one *Scenario* 4.

For *Scenario 3*, the posterior gene selection probabilities, averaged over 100 sets of simulated data are shown in the top part of Figure 4. All the casual genes (marked by red color) still show significantly higher posterior selection probabilities than other genes. This further verified that our iBVS method works well for the case with p = 2000 genes, a number that we believe is commonly encountered in practical applications, as the majority of genes are unchanged between conditions or expressed at baseline levels. Compared to the simulation result with p = 315 genes, we found that the posterior gene selection probabilities are much lower in the case of p = 2000. For example, the selection probability for the causal gene 95, reduced from 93.6% (when p = 315) to 72.4% (when p = 2000).

In *Scenario 4*, the bottom figure in Figure 4 shows that all the casual genes (marked by red color) still show notably higher posterior selection probabilities than other non-causal genes. But this time, the cutoff between causal and non-causal gene selection is not as clear as in *Scenario 3*. It is interesting to see that the posterior selection probabilities are not that high for the 3rd, 4th, and 5th causal gene groups. This is because the groups associate with genes that are not connected to each other (in other words, they independently influence the phenotype or disease status). When groups of highly correlated causal genes are working in concert, they jointly show higher impact to the phenotype or disease status.

#### Determine significant causal or marker genes

**Figure 5:**
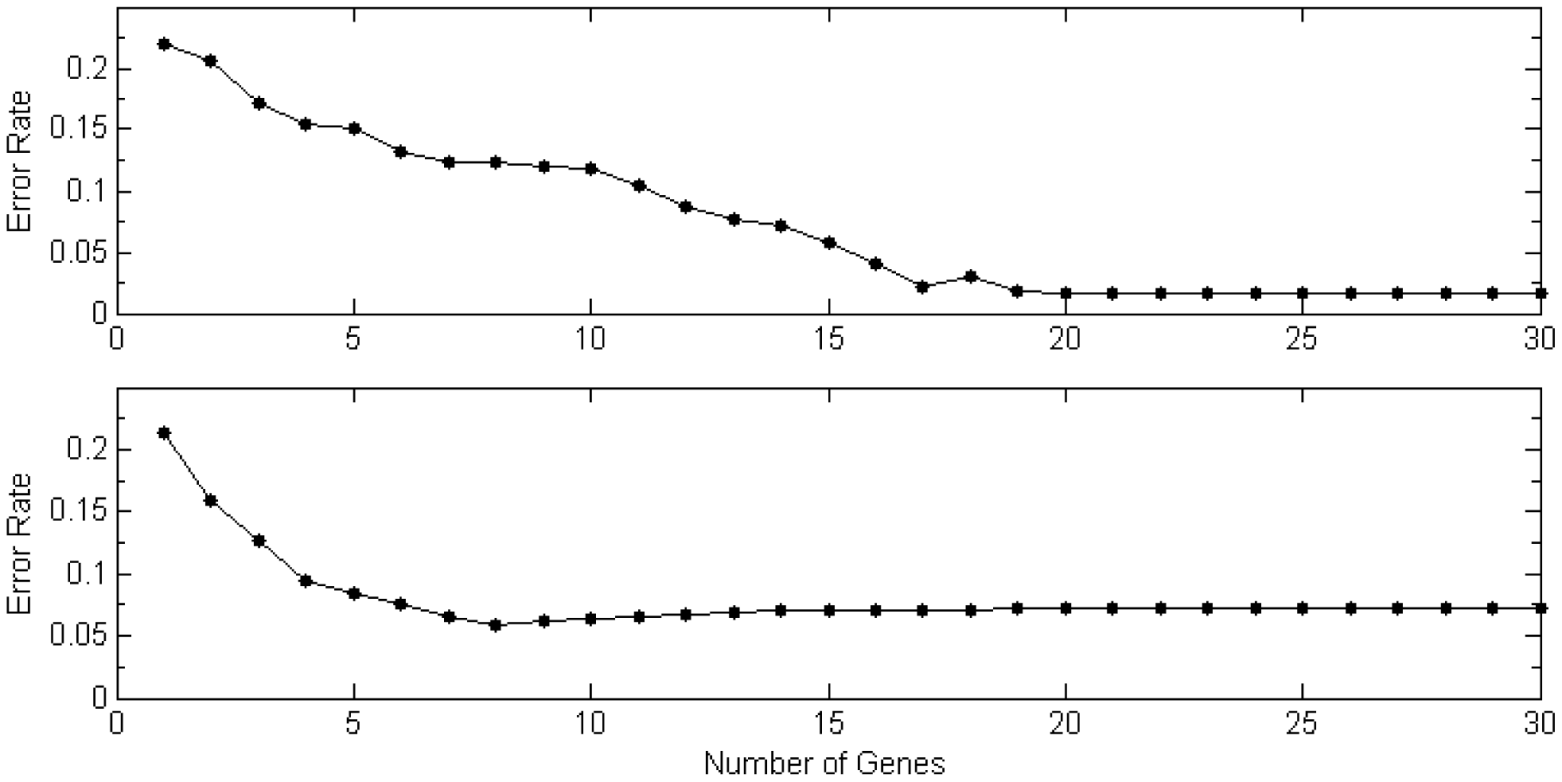
Mean Square Error for Gene Selections. Averaged over 100 simulated data in *Scenario* 1 for two set of gene effect sizes 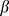. The top one is for 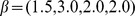 and bottom one 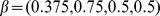.

When we determine which or how many genes are significant causal or marker genes based on the posterior probabilities distribution of all genes, we use cross-validation methods. In this procedure, a logistic regression model was used to examine the relationship between genes and the binary outcome variable. We started from simplest logistic regression model only including the gene with the highest posterior probability. Then we add the gene with next highest posterior probability to the model one at a time, until reaching a total number of 30 genes included in model. Two datasets were chosen randomly from 100 datasets, with one being used for estimating the regression coefficients of the model, and the other estimating the prediction error. We repeated this 200 times to find the average predicting error. The results of average prediction error are shown in Figure 5 for 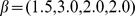 and 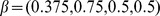. It was clear in the first plot that the model including the best eight genes had the lowest prediction error, where the eight genes were exactly the same simulated causal genes. In the second plot when 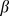 was smaller, we saw that the model with 17 genes performed the best. Note that among the 17 genes 13 are causal genes and 4 are non-causal genes.

#### Compare iBVS with standard BVS

We further verified the advantage of our iBVS method with informative priors constructed from known gene-gene networks or pathways. To do this, we compared our method with other standard BVS schemes without informative priors. The method of Yang & Song [70] represents such a standard BVS method, (will be referred as YS-BVS later on), which is also the most comparable method to ours. In YS-BVS procedure, pathway selection is not considered and the existence of network relationships between the genes was completely ignored.

**Figure 6:**
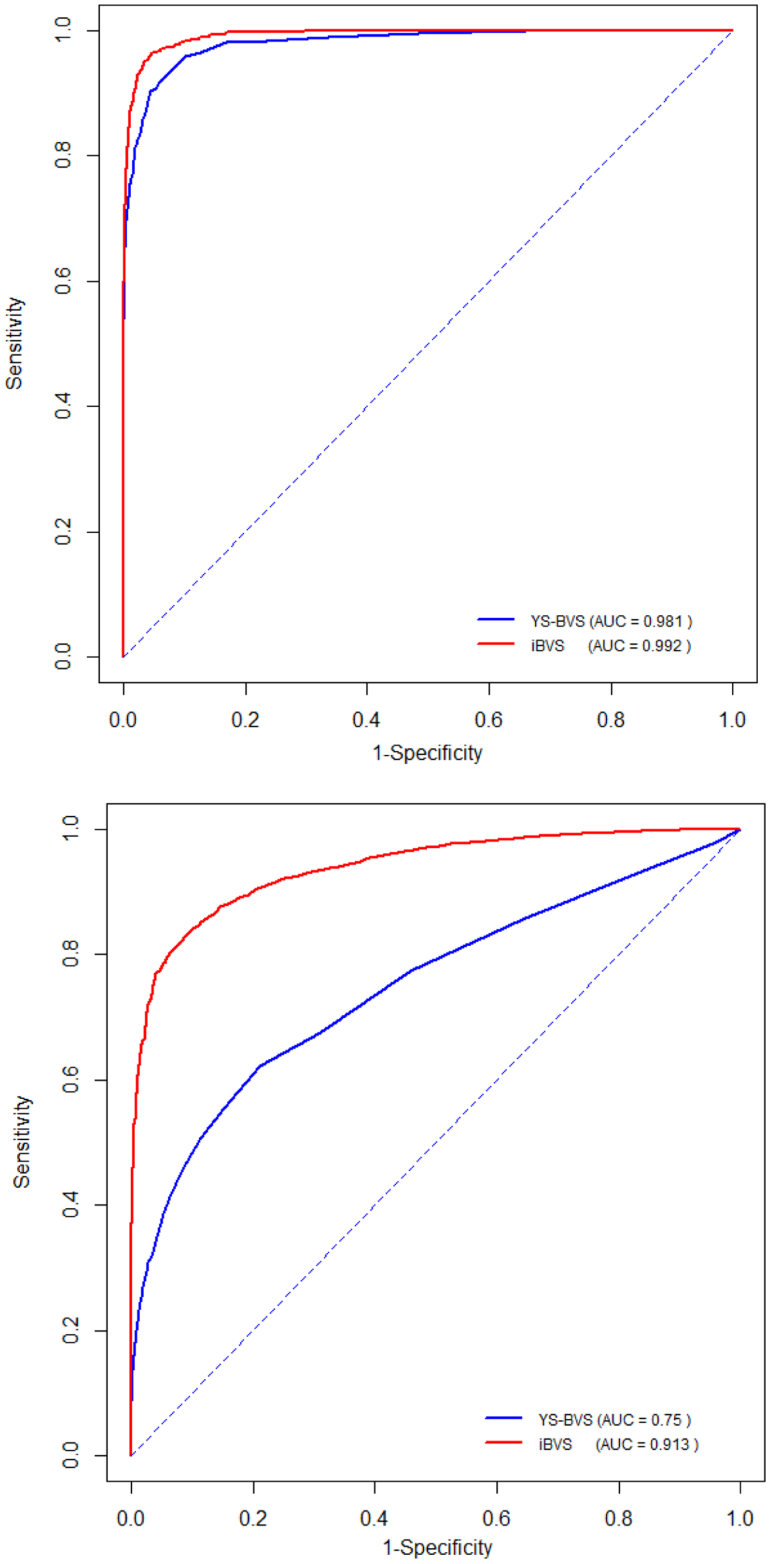
ROC Curves for iBVS and YS-BVS (Yang & Song's BVS).

We ran YS-BVS to the same sets of simulated data. The following ROC curves for gene selection in two scenarios provide a direct comparison of this method with ours on gene selection accuracy in terms of sensitivity and specificity. From the plots in Figure 6, it is obvious that both in the case of small number causal genes (*Scenario 1*) and in the case of large number small-effect genes (*Scenario 2*), our method has notably larger AUC (area under curve). For example, the AUC is 0.992 for iBVS compared to 0.981 for YS-BVS in Scenario 1. This is especially true for *Scenario 2* (AUC = 0.913 for iBVS and 0.750 for YS-BVS), which suggests that in dealing with diseases of complete genomic mechanisms involving many tiny-effect causal/marker genes, to consider gene selection within the given network/pathway background would definitely be a better approach for the task of biomarker identification. And when applying both methods to simulated data with higher noise levels (see Figure 6), our iBVS has a greater and significant advantage over YS-BVS.

### Application

A blood-based biomarker of acute ischemic stroke is of significant value in clinical practice. Deidentified data was used from consented subjects recruited as part of the CLEAR Trial from the University of Cincinnati (Pancioli et al. [85]). Ischemic stroke was scored by clinical evaluation and evident by neuroimaging. Demographic information for stroke subjects and healthy volunteers was recorded. Blood samples were drawn into PAXgene tubes (PreAnalytiX, Hilden, Germany) within three hours of stroke onset and prior to administration of any medication. RNA was isolated, prepared and hybridized to Affymetrix Human U133 Plus 2.0 microarrays as previously described (Stamova et al. [86]) This study aimed to (1) identify genes in differentiating stroke patients (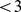-hr after stroke) from healthy controls; and (2) identify pathways as groups of genes in differentiating stroke patients from controls.

### Analysis Procedure

A 2-step STS strategy for biomarker identification was adopted in this application. Firstly, a robust gene screening and pathway analysis was conducted; then followed by the conduct of simultaneously selection of genes and pathways using the proposed iBVS method.

We first selected 815 probe sets by using univariate t-test (genefilter R package; rowttest) at significance level 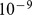. These probe sets correspond to 605 unique genes. By mapping these genes to the KEGG database, we found 163 pathways, each containing at least one of the 605 genes. These 163 pathways contained 5467 genes in total. This group of genes was referred as *grand signature gene set* and it contained too many candidate genes for our iBVS discovery procedure.

To further reduce the number of candidate genes, we considered two schemes. The first one was by conducting gene set enrichment analysis (GSEA) based on the hypergeometric distribution [40]. In this GSEA, each of the 163 pathways was viewed as a gene set and the network topology was totally ignored. By setting the p-value cut-off of 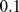, we kept 24 pathways for the following iBVS analysis; all with 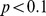. These 24 pathways contained a total of 1216 genes. For reference, these genes together is termed *Signature Gene Set*. An alternative approach is to subjectively select a small number of pathways according to their known biological functions that are related to stroke or cardiovascular problems. This method was not applied because, unlike protein-protein interaction networks, KEGG pathways offer less clinical interpretation.

Since we only have microarray data defined on probe set level, a procedure of mapping the probe sets to genes was also needed. We followed the lead of Li et al. [87] to choose only one probe set to represent the expression level of a gene. If multiple probe sets were mapped to one gene, we kept the one with smallest 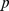 value in the above multiple t-test procedure.

Finally we conducted the iBVS analysis with PLS g-prior by considering only the Signature Gene Set and the associated 24 KEGG pathways. Then we followed the iBVS method for binomial regression with Probit distribution to carry out the variable selection. Similar hyper-parameters were set as in the simulation studies and we used Gelman and Rubin diagnostics [72] to determine the burn-in length as 10000 iterations and 50000 additional iterations were run to make posterior inferences. It took 5 hours and 40 minutes using a desktop computer with single core 4.5GHz CPU and 4GB memory.

### Application Results

**Figure 7:**
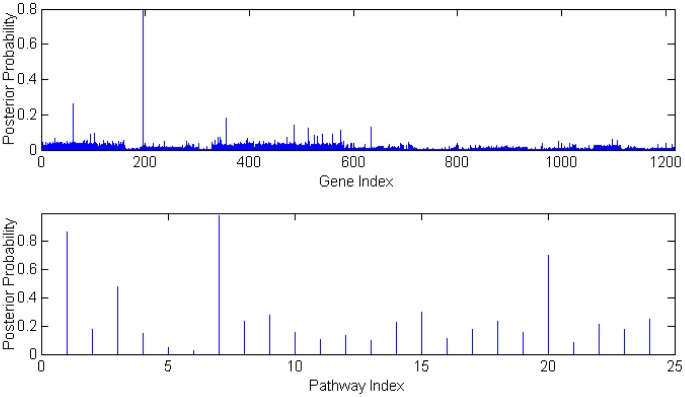
Gene and Pathway Selection Results for Stroke Data.

Figure 7 shows the posterior probabilities of genes selected via our iBVS strategy with integrated biological priors. The top 30 genes (probe sets) are listed in Table 1. In order to select only the most efficient predictive genes, cross-validation for our iBVS model was used. The top genes were added into the logistic model, one by one, to estimate the prediction error. The error analysis of the model with inclusion of different numbers of predictive genes shows that the smallest classification error appears when only the top 3 or 4 genes are selected as predictors. The error increases with the number of predictors of more than 4, but the errors greatly decrease again when the 13th or 16th genes are included in the model. The top 5 pathways are listed in Table 2.

**Table 1:** Top 30 genes selected using BVS on Stroke Data.

No	BVS.ID	Post.Prob.	Probe.Set.ID	Gene.Symbol	Gene.Title
1	196	0.951	206177_s_at	ARG1	arginase, liver
2	61	0.26	202635_s_at	POLR2K	polymerase (RNA) II (DNA directed) polypeptide K, 7.0kDa
3	356	0.184	205067_at	IL1B	interleukin 1, beta
4	486	0.15	1552912_a_at	IL23R	interleukin 23 receptor
5	634	0.126	235086_at	THBS1	thrombospondin 1
6	514	0.125	207445_s_at	CCR9	chemokine (C-C motif) receptor 9
7	576	0.114	207113_s_at	TNF	tumor necrosis factor
8	103	0.096	203939_at	NT5E	5'-nucleotidase, ecto (CD73)
9	541	0.091	206126_at	CXCR5	chemokine (C-X-C motif) receptor 5
10	95	0.087	219308_s_at	AK5	adenylate kinase 5
11	559	0.085	214146_s_at	PPBP	pro-platelet basic protein (chemokine (C-X-C motif) ligand 7)
12	524	0.082	210549_s_at	CCL23	chemokine (C-C motif) ligand 23
13	339	0.076	205291_at	IL2RB	interleukin 2 receptor, beta
14	530	0.074	216598_s_at	CCL2	chemokine (C-C motif) ligand 2
15	472	0.071	205445_at	PRL	prolactin
16	343	0.069	207072_at	IL18RAP	interleukin 18 receptor accessory protein
17	26	0.067	223359_s_at	PDE7A	phosphodiesterase 7A
18	397	0.066	211333_s_at	FASLG	Fas ligand (TNF superfamily, member 6)
19	1098	0.059	52255_s_at	COL5A3	collagen, type V, alpha 3
20	394	0.058	241819_at	TNFSF8	tumor necrosis factor (ligand) superfamily, member 8
21	89	0.056	212739_s_at	NME4	non-metastatic cells 4, protein expressed in
22	158	0.056	203302_at	DCK	deoxycytidine kinase
23	334	0.055	205327_s_at	ACVR2A	activin A receptor, type IIA
24	448	0.054	210755_at	HGF	hepatocyte growth factor (hepapoietin A; scatter factor)
25	119	0.054	205757_at	ENTPD5	ectonucleoside triphosphate diphosphohydrolase 5
26	346	0.053	205403_at	IL1R2	interleukin 1 receptor, type II
27	344	0.053	206618_at	IL18R1	interleukin 18 receptor 1
28	1107	0.053	204614_at	SERPINB2	serpin peptidase inhibitor, clade B (ovalbumin), member 2
29	560	0.052	215101_s_at	CXCL5	chemokine (C-X-C motif) ligand 5
30	80	0.051	1553587_a_at	POLE4	polymerase (DNA-directed), epsilon 4 (p12 subunit)

We list the detailed information on the top 30 genes. BVS.ID refers to the variables in the model: e.g. 196 refers to 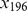 in our model. Post.Prob. is the posterior probability of the particular variable.

**Table 2:** Top Pathways Selected via BVS.

No	KEGG.ID	Name	Top.genes.extracted	Total # of genes
1	Hsa05214	Glioma - Homo sapiens (human)	BVS.ID356 (IL1B), BVS.ID486 (IL23R)	253
2	Hsa04060	Cytokine-cytokine receptor interaction- Homo sapiens (human)	BVS.ID61 (POLR2K)	160
3	Hsa05222	Small cell lung cancer - Homo sapiens (human)	BVS.ID196 (ARG1)	106
4	Hsa04623	Cytosolic DNA-sensing pathway- Homo sapiens (human)	BVS.ID196 (ARG1)	55
5	Hsa04640	Hematopoietic cell lineage - Homo sapiens (human)		107

We list the 5 pathways that have the highest posterior probabilities. Top.genes.extracts refers to the gene with highest posterior probability within a pathway. and Total # of genes refers to the total number of genes within a pathway.

From a biological standpoint, the genes and pathways that represent the best gene predictors and key pathways are directly relevant. ARG1, the gene with the greatest predictive value, is a marker of M2 macrophage activation (Morris et al. [88]), which is associated with the inflammatory and immune response to stoke. Abrupt changes in gene transcription triggered as a response to stroke for initiation of cellular survival mechanisms would be mediated through POLR2K as a pivotal player in RNA transcription. IL1B and IL23R are also amongst the other top genes with the greatest predictive value. These and many others identified in Table 1 are key modulators in effecting the inflammatory response of cells responding to the injury of stroke (Wong et al. [89]).

Given the abundance of chemokine and immune modulating genes in our list, it was not surprising to see that the KEGG pathway for cytokine-cytokine interaction is represented, as is the highly immune pathway for cytosolic DNA-sensing. While gliomas are brain-related like stroke, the presence of this pathway may represent its more general cytokine or calcium-related signaling features. Smaller overlapping sub-networks of characterized pathways may account for the presence of seemingly unrelated, yet identified pathways such as that for small cell lung cancer, as it contains key components of cell proliferation and cell death, which are also prominent in brain injury.

## Discussion

In this paper, we used a generalized Bayesian framework for biomarker identification. For problems with 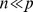, it would be appealing to remove noisy measures or those with lower quality beforehand and defining the proper level of model space to be further explored using stochastic search. We then followed the integrative biomarker discovery scheme to incorporate the gene network, i.e. pathway information, and adopted a novel PLS g-prior for the purpose of variable selection. Cross-validation methods were conceived for determining the Bayesian significance level in cutting off the posterior probabilities for selecting causal/marker genes in classifying patients or predicting risk of diseases.

### Subjective versus Objective Priors

In this paper, we mainly adopt the perspective of subjective Bayesian due to the fact that we want to incorporate informative priors from available scientific sources. Although we used MRF in this article to illustrate how gene-gene networking structure would cast upon gene selection, there are many different ways to use the abundant informative priors (Hill et al. [75]). As seen in the Method section, even for MRF, we have different ways to incorporate this information. Choosing an objective prior that satisfies some fundamental principles as summarized in Bayarri et al. [52] would be theoretically appealing. For example, when specifying the prior distribution of the gene/pathway selection probability, we may choose a Bernoulli distribution with unknown hyper-parameter 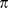 with Beta prior distribution, instead of setting it at a fixed level. This would lead to the posterior selection of models that are not biased toward a mode dimension of 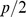.

### Comparison to Other Marker Discovery Methods

As mentioned above briefly, regularization methods provide an alternative solution for feature selection and classification problems. For GWAS data, Guan and Stephens [4] have indicated that BVS provides better power and predictive performance than standard lasso techniques. Our experience with standard BVS for simulated microarray data with continuous outcomes also suggest that it outperforms lasso, elastic net [90], and stepwise variable selection with higher sensitivity and specificity. Nonetheless, there lacks of evidence in comparing BVS with grouped lasso [91], which considers the grouping of genes into gene sets. As proved in the simulation studies, our iBVS performs better than, or at least equally as well as standard BVS for gene selection. It also has the advantage to tell you which networks, in addition to which genes, could predict disease and pathology. Compared to network-based marker discovery, our iBVS not only suggests which genes are important, but also could handle those ‘orphan’ genes that have not been classified into any pathway at the time of study.

In the conduct of standard GSEA or network marker discovery, one may calibrate the significance of a pathway in predicting disease or treatment effectiveness by using all its predefined member genes, but these include a large number of noisy genes (i.e., those non-contributive and non-causal genes). Alternatively, one may choose to use only a subset of contributing genes, but which subset to use is a big challenge. In our iBVS, the two components are merged together into one procedure, which allows the two parts learn from each other and reflects the uncertainty of gene/pathway selection using stochastic simulation.

### Future Directions

Although iBVS has been proven as an appealing alternative solution to traditional gene-wise biomarker identification, its computational challenges hinder its widespread adoption. With a large number of parameters in the model, the inference is mainly based on Monte Carlo simulation, which is time-consuming. Running over single computers, it would take hours even days to complete a round of simulation procedure. Nowadays, with the advent of high-speed cluster computers and the existence of cloud computing technologies, it is becoming very feasible to apply full iBVS methods for biomarker identification. Our research team is developing parallel MCMC algorithm over the Amazon Cloud platform using the idea of MapReduce.

Currently, the pathway information we have is limited to a small portion of genes that have been well-characterized. A relatively large amount of genes are not well-studied, nor their functions have been identified. In our application, we found that some genes had not been mapped to any KEGG pathways yet. Two potential solutions are conceived: (1) develop a stochastic inference of the gene-gene networks from the data and merge it into the current BVS MCMC algorithm; (2) query the Internet to find as more information, literature, and databases to help elicit richer priors. This topic is part of our ongoing research.

## Supporting Information

Text S1 (PDF)
