# Supplementary material for: An Integrative Framework for Bayesian Variable Selection with Informative Priors for Identifying Genes and Pathways
Source: PLoS One. 2013 Jul 3;8(7):e67672. doi: 10.1371/journal.pone.0067672 (PMC3700986; doi:10.1371/journal.pone.0067672)
Supplement: Text S1 — (PDF) [file pone.0067672.s001.pdf]

An Integrative Bayesian Hierarchical Modeling Framework for Gene and Pathway Identification with Informative Priors

Bin Peng, Dianwen Zhu, Bradley P. Ander, Xiaoshuai Zhang, Fuzhong Xue, Frank R. Sharp, Xiaowei Yang

Supplement Text S1:

Derivation of $p(Z, \xi, \gamma | Y, \mathbf{X})$:

We have the following distributions for priors α, β:

$$\alpha \sim \mathcal{N}(0, h), \quad \beta_{(\xi,\gamma)}|(\xi, \gamma) \sim \mathcal{N}(\mathbf{0}, c(\mathbf{T}'_{(\xi,\gamma)}\mathbf{T}_{(\xi,\gamma)})^{+})$$

Then since

$$\begin{aligned} Z_i &= \alpha + T_{i,(\xi,\gamma)}\beta_{(\xi,\gamma)} + \epsilon_i, \\ \epsilon_i &\sim \mathcal{N}(0,1), \quad i = 1, \cdots, n, \end{aligned} \tag{1}$$

we have

$$Z_i|Y, \mathbf{X}, \alpha, \beta_{(\xi,\gamma)}, \xi, \gamma \sim \mathcal{N}(\alpha + \mathbf{T}_{i(\xi,\gamma)}\beta_{(\xi,\gamma)}, 1)I(A_i)$$

where A_i corresponds to $\{Z_i : Z_i > 0\}$ and $\{Z_i : Z_i \leq 0\}$ respectively; and $I(\cdot)$ is the indicator function which truncates the univariate normal distribution of Z_i to the appropriate region.

The joint posterior distribution of $(Z, \alpha, \beta_{(\xi,\gamma)}, \xi, \gamma)$ given $(Y, \mathbf{X})$ is

$$\begin{aligned} p(Z, \alpha, \beta_{(\xi,\gamma)}, \xi, \gamma|Y, \mathbf{X}) \propto& \prod_{i=1}^{n} p(Z_i|Y, X, \alpha, \beta_{(\xi,\gamma)}, \xi, \gamma)p(\alpha)p(\beta_{(\xi,\gamma)}|\mathbf{X}, \xi, \gamma)p(\xi, \gamma) \\ \propto& [\exp(-\frac{\sum_{i=1}^{n}(Z_i - \alpha - (\mathbf{T}_{(\xi,\gamma)}\beta_{(\xi,\gamma)})_i)^2}{2})\prod_{i=1}^{n} I(A_i)] \times \exp(-\frac{\alpha^2}{2h}) \\ &\times [\exp(-\frac{\beta'_{(\xi,\gamma)}\mathbf{T}'_{(\xi,\gamma)}\mathbf{T}_{(\xi,\gamma)}\beta_{(\xi,\gamma)}}{2c})\prod_{i=1}^{m_\xi}\lambda_i^{-\frac{1}{2}}] \times p(\xi, \gamma) \end{aligned} \tag{2}$$

where $\lambda_1, \cdots, \lambda_{m_\xi}$, $(m_\xi \leq p_\xi)$ are the nonzero eigenvalues of $(\mathbf{T}'_{(\xi,\gamma)}\mathbf{T}_{(\xi,\gamma)})^{+}$.

We first integrate α out given $Z, \beta_{(\xi,\gamma)}, \xi, \gamma$. We rewrite the exponents for the terms that have α involved in (2) as:

$$
\begin{aligned}
&-\frac{\sum_{i=1}^{n}(Z_i - \alpha - \mathbf{T}_{i(\xi,\gamma)}\beta_{(\xi,\gamma)})^2}{2} - \frac{\alpha^2}{2h} \\
=&-\frac{(n+h^{-1})\alpha^2 - 2\alpha\mathbf{1}'(Z - \mathbf{T}_{(\xi,\gamma)}\beta_{(\xi,\gamma)}) + (Z - \mathbf{T}_{(\xi,\gamma)}\beta_{(\xi,\gamma)})'(Z - \mathbf{T}_{(\xi,\gamma)}\beta_{(\xi,\gamma)})}{2} \\
=&-\frac{(n+h^{-1})(\alpha - (n+h^{-1})^{-1}\mathbf{1}'(Z - \mathbf{T}_{(\xi,\gamma)}\beta_{(\xi,\gamma)}))^2}{2} - \\
&-\frac{(Z - \mathbf{T}_{(\xi,\gamma)}\beta_{(\xi,\gamma)})'(I_n - (n+h^{-1})^{-1}\mathbf{1}\mathbf{1}')(Z - \mathbf{T}_{(\xi,\gamma)}\beta_{(\xi,\gamma)})}{2}
\end{aligned}
\tag{3}
$$

We can see that the first term in (3) can be integrated out by the property of a Guassian kernel, then we only have the second term left to be combined with other terms involving $\beta_{(\xi,\gamma)}$.

Before proceeding, we notice that

$$
I_n - (n+h^{-1})^{-1}\mathbf{1}\mathbf{1}' = (I_n + h\mathbf{1}\mathbf{1}')^{-1},
$$

which can be straightforwardly verified by hand. Next we expand the 2nd term in (3) in terms of $\beta_{(\xi,\gamma)}$ and then combine with the 4th term in (2) and rewrite the result as:

$$
\begin{aligned}
&-\frac{\beta'_{(\xi,\gamma)}\mathbf{T}'_{(\xi,\gamma)}(I_n + h\mathbf{1}\mathbf{1}')^{-1} + c^{-1}I_n)\mathbf{T}_{(\xi,\gamma)}\beta_{(\xi,\gamma)} - 2\beta'_{(\xi,\gamma)}\mathbf{T}'_{(\xi,\gamma)}(I_n + h\mathbf{1}\mathbf{1}')^{-1}Z}{2} \\
&\qquad -\frac{Z'(I_n + h\mathbf{1}\mathbf{1}')^{-1}Z}{2} \\
=&-\frac{(\beta_{(\xi,\gamma)} - A^{-1}B)'A(\beta_{(\xi,\gamma)} - A^{-1}B)}{2} - \frac{Z'(I_n + h\mathbf{1}\mathbf{1}')^{-1}Z - B'A^{-1}B}{2}
\end{aligned}
\tag{4}
$$

where $A = \mathbf{T}'_{\xi}((I_n + h\mathbf{1}\mathbf{1}')^{-1} + c^{-1}I_n)\mathbf{T}_{(\xi,\gamma)}, \quad B = \mathbf{T}'_{(\xi,\gamma)}(I_n + h\mathbf{1}\mathbf{1}')^{-1}Z$. Note that we'll use the Moore-Penrose pseudo-inverse of A shall it be non-invertible.

Again here we have completed a quadratic form in $\beta_{(\xi,\gamma)}$, which can be integrated out, and then we examine the full form of the 2nd term in (4):

$$
\begin{aligned}
-&\frac{Z'\{(I_n + h\mathbf{1}\mathbf{1}')^{-1} - (I_n + h\mathbf{1}\mathbf{1}')^{-1}\mathbf{T}_{(\xi,\gamma)}}{2} \\
&\qquad \frac{[\mathbf{T}'_{(\xi,\gamma)}((I_n + h\mathbf{1}\mathbf{1}')^{-1} + c^{-1}I_n)\mathbf{T}_{(\xi,\gamma)}]^{-1}\mathbf{T}'_{(\xi,\gamma)}(I_n + h\mathbf{1}\mathbf{1}')^{-1}\}Z}{2}.
\end{aligned}
\tag{5}
$$

Taking $A = I_n + h\mathbf{1}\mathbf{1}'$, $B = c(\mathbf{T}'_{(\xi,\gamma)}\mathbf{T}_{(\xi,\gamma)})^{+}$, $U = \mathbf{T}_{(\xi,\gamma)}$, $V = \mathbf{T}'_{(\xi,\gamma)}$ and applying the Woodbury matrix inversion lemma (again, we will adopt the Moore-Penrose pseudo-inverse wherever necessary):

$$
(A + UBV)^{-1} = A^{-1} - A^{-1}U(B^{-1} + VA^{-1}U)^{-1}VA^{-1}
$$

we immediately see that (5) equals

$$-\frac{Z'\Sigma_{(\xi,\gamma)}^{-1}Z}{2} \quad \text{with}\, \Sigma_{(\xi,\gamma)} = I_n + h\mathbf{1}\mathbf{1}' + c\mathbf{T}_{(\xi,\gamma)}(\mathbf{T}'_{(\xi,\gamma)}\mathbf{T}_{(\xi,\gamma)})^{+}\mathbf{T}'_{(\xi,\gamma)}.$$

Since we also have priors on (ξ, γ) as

$$p(\xi,\gamma) \propto \prod_{k=1}^{K_\xi} \pi_k^{\theta_k}(1-\pi_k)^{1-\theta_k} \exp(\mu\mathbf{1}'_p\gamma + \eta\gamma'\mathbf{R}\gamma)$$

we finally obtain

$$\begin{aligned} p(Z,\xi,\gamma|Y,\mathbf{X}) \quad \propto \quad & \frac{1}{|\Sigma_{(\xi,\gamma)}|^{\frac{1}{2}}} \exp(-\frac{Z'\Sigma_{(\xi,\gamma)}^{-1}Z}{2}) \prod_{i=1}^{n} I(A_i) \\ & \times \prod_{k=1}^{K_\xi} \pi_k^{\theta_k}(1-\pi_k)^{1-\theta_k} \exp(\mu\mathbf{1}'_p\gamma + \eta\gamma'\mathbf{R}\gamma). \qquad \square \end{aligned}$$
